# Effect of a multi-component enhanced recovery after surgery integrated protocol based on preoperative risk stratification and dynamic rehabilitation adjustment on functional recovery after knee and hip arthroplasty: a retrospective cohort study

**DOI:** 10.3389/fmed.2026.1836698

**Published:** 2026-10-07

**Authors:** Xiaolong Ma, Kai Zhang, Wenbin Yang, Zirui Zhang, Xiuling Li

**Affiliations:** 1 Rehabilitation Medicine Department, Joint Logistics Support Force, People’s Liberation Army, No. 940 Hospital, Lanzhou, China; 2 Department of Orthopedics, Joint Logistics Support Force, People's Liberation Army, No. 989 Hospital, Luoyang, China

**Keywords:** accelerated rehabilitation surgery, ERAS, hip replacement, knee replacement, smart devices

## Abstract

**Objective:**

To investigate the impact of a multicomponent enhanced recovery after surgery (ERAS) protocol based on preoperative risk stratification and dynamic rehabilitation adjustment on functional recovery following total knee arthroplasty (TKA) or total hip arthroplasty (THA).

**Methods:**

A retrospective cohort study was conducted, enrolling 256 patients who underwent primary unilateral TKA or THA at the Ninth Medical Center of the Joint Logistics Support Force of the People's Liberation Army between September 2023 and September 2024. Patients were divided into a study group (*n* = 128) receiving an ERAS protocol guided by rehabilitation assessment and a control group (*n* = 128) receiving a conventional ERAS protocol. The study group underwent preoperative risk stratification using a predictive model (low, medium, high) and received individualized, stepwise rehabilitation training based on daily activity load monitored via wearable devices; the control group followed a standardized, uniform rehabilitation protocol. Primary outcome measures included Visual Analog Scale (VAS) scores preoperatively and postoperatively at 1 day, 3 days, 1 month, 3 months, and 6 months. HSS scores were used for TKA patients and Harris scores for THA patients. Generalized estimating equations were applied to adjust for baseline confounding factors. Incidences of deep vein thrombosis (DVT) and surgical site infection (SSI) were also recorded.

**Results:**

After adjusting for baseline confounders, the intervention group had significantly lower VAS pain scores across all postoperative follow-up time points (*P* < 0.05). For TKA patients, the intervention group showed significantly higher HSS scores than the control group starting from postoperative day 3 (*P* < 0.05): the control group had no significant change in HSS score on postoperative day 3 vs. baseline (*P* > 0.05), while the intervention group achieved significant improvement at the same time point (*P* < 0.05). For THA patients, the intervention group had significantly higher Harris hip function scores than the control groups starting from postoperative day 3 (*P* < 0.05): the control group showed no significant difference in Harris score on postoperative day 3 vs. baseline (*P* = 0.069), with significant improvements observed at 1, 3, and 6 months postoperatively (*P* < 0.05). No significant intergroup differences were found in the incidences of DVT and SSI in either the TKA or the THA subgroups (*P* > 0.05).

**Conclusion:**

The multicomponent ERAS protocol incorporating preoperative risk stratification and dynamic rehabilitation adjustment is significantly associated with improved short-term pain relief and functional recovery after TKA and THA. A prominent functional improvement trend was observed, especially during the critical early rehabilitation window at 1-month post-arthroplasty. The intergroup differences in both HSS and Harris Hip scores peaked at 3 months postoperatively. These findings provide real-world observational evidence supporting precision rehabilitation following joint arthroplasty.

## 1 Introduction

Enhanced recovery after surgery (ERAS) refers to the implementation of evidence-based rational perioperative interventions designed to alleviate the postoperative stress response, reduce surgical trauma, relieve postoperative pain, lower the incidence of complications, accelerate functional recovery, improve patient satisfaction, and shorten the overall rehabilitation process (1). In recent years, ERAS protocols have received extensive attention and have been widely promoted in domestic orthopedics, challenging numerous traditional treatment and management concepts and achieving favorable clinical outcomes (2). However, challenges such as significant individual variability and insufficient standardization in postoperative functional recovery remain (3, 4). Most existing ERAS studies adopt a uniform standardized rehabilitation pathway, ignoring the individual differences in patients' preoperative baseline status and rehabilitation tolerance. Although several studies have proposed risk stratification and wearable device monitoring in arthroplasty rehabilitation, few have integrated these components into a complete perioperative ERAS pathway, and real-world retrospective cohort data are lacking to verify the association between this integrated protocol and short-term postoperative functional outcomes. This study integrated a bundled ERAS intervention protocol consisting of a self-developed risk prediction model, dynamic load monitoring via smart wristbands, and gradient rehabilitation training. A historical controlled cohort analysis was conducted to explore its correlation with postoperative pain and joint functional indicators after total knee arthroplasty (TKA)/total hip arthroplasty (THA), providing real-world observational evidence to guide precise arthroplasty rehabilitation.

## 2 Materials and methods

Inclusion Criteria: (1) Age ranging from 45 to 80 years; (2) Underwent primary unilateral TKA or THA; (3) All surgical procedures were performed by experienced attending joint surgeons; (4) The study obtained full consent from both the patients themselves and their family members, with written informed consent duly signed, and the entire protocol was reviewed and approved by the institutional ethics committee.

Exclusion Criteria: (1) Complicated by severe cardiopulmonary dysfunction or coagulation disorders; (2) Previous surgical history on the ipsilateral limb or prior revision arthroplasty history; (3) Missing of core rehabilitation assessment data or key follow-up endpoints in archived medical records; (4) Loss to follow-up before the study reached its predefined endpoint.

This study was designed as a retrospective cohort study. Patient allocation between the two groups was determined by the perioperative management protocol implemented in routine clinical practice: patients admitted from September 2023 to March 2024 received the conventional ERAS protocol (traditional group, *n* = 128), while patients admitted from April 2024 to September 2024 received the multicomponent assessment-based ERAS protocol (study group, *n* = 128). Standardized functional outcome scores collected from preoperation to 6 months postoperatively was designated as the primary analysis endpoints, and only two adverse events—deep vein thrombosis (DVT) and surgical site infection (SSI)—were recorded for the period from 6 months post-operation to the final follow-up. All surgeries for patients in both groups were completed by the same fixed team of joint surgeons, and all perioperative nursing procedures were carried out in the identical inpatient nursing unit, to minimize the confounding effects of provider variability on clinical outcomes (Figure 1).

**Figure 1:**
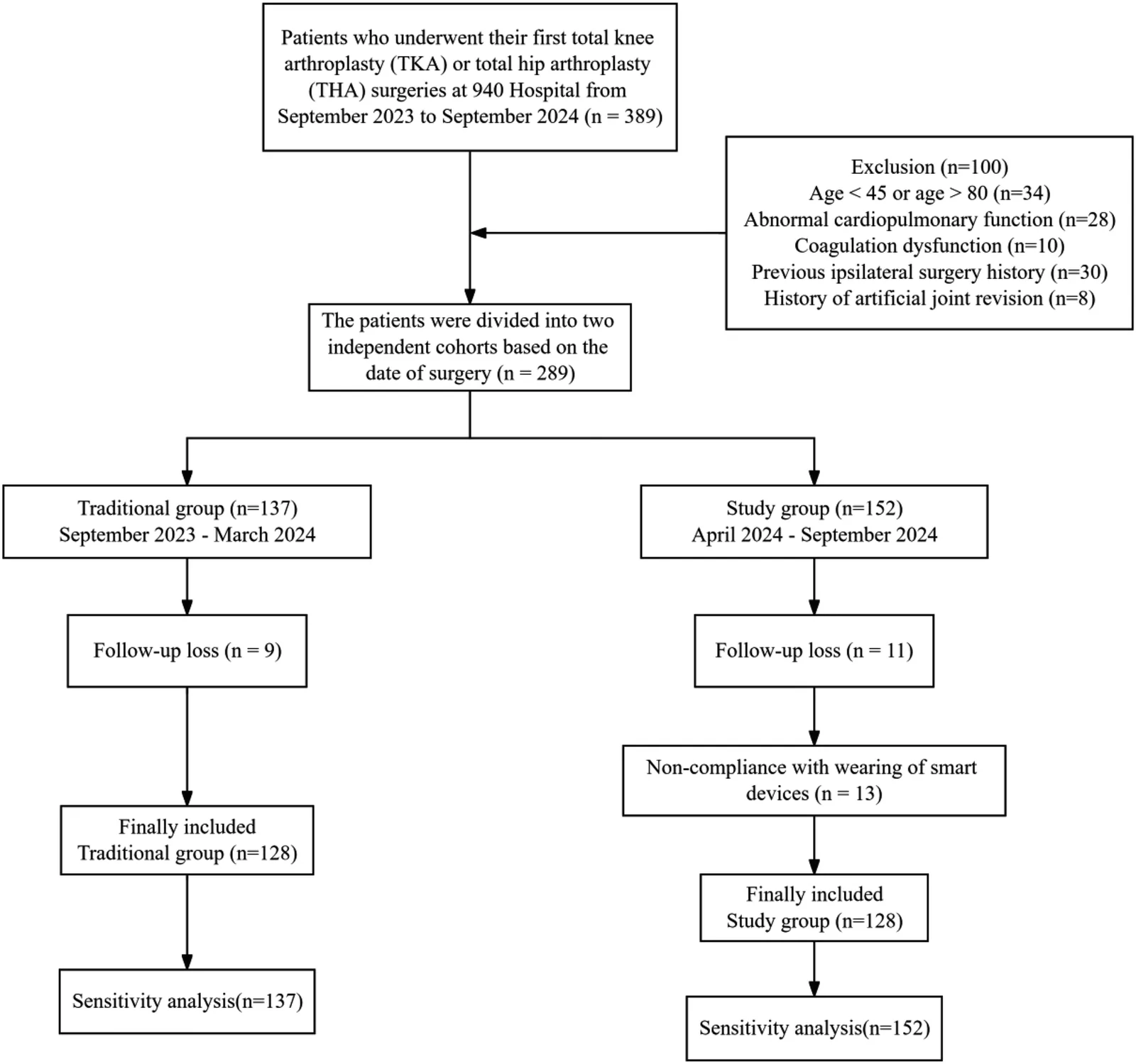
Flowchart of research screening process.

At the time of enrollment, baseline data were collected for all patients, including demographic characteristics, lifestyle factors, comorbid chronic diseases, preoperative treatment history, anesthesia regimen, surgery-related parameters, and preoperative pain and joint function scores. Baseline comparisons between the study group and the control group were performed separately within the TKA subgroup and the THA subgroup (Tables 1, 2). To capture complete information on long-term postoperative complications, all postoperative adverse events were continuously recorded during standardized telephone follow-ups, with all final outcomes verified against electronic medical records; the maximum follow-up duration was 12–17 months after surgery. All DVT diagnoses were confirmed by lower-extremity venous color Doppler ultrasonography (5), and SSI was defined according to criteria established by the US Centers for Disease Control and Prevention (CDC) (6).

**Table 1:** Baseline data of knee joint.

Parameters		Study group	Traditional group	*t*/*Z*/*χ*^2^	*P*
Sex *n* (%)	M	31 (48.4%)	27 (42.2%)	0.504	0.478
	F	33 (51.6%)	37 (57.8%)		
Age (year, *x¯* ± *s*)		55.190 ± 4.666	55.230 ± 4.563	−0.057	0.954
BMI (kg/m^2^, *x¯* ± *s*)		25.520 ± 3.157	26.055 ± 3.111	−0.965	0.337
Smoking *n* (%)	Yes	10 (15.6%)	6 (9.4%)	1.143	0.285
	No	54 (84.4%)	58 (90.6%)		
Drinking *n* (%)	Yes	8 (12.5%)	4 (6.3%)	1.471	0.225
	No	56 (87.5%)	60 (93.8%)		
Intra-articular injection/acupuncture *n* (%)	Yes	3 (4.7%)	11 (17.2%)	5.133	0.023
	No	61 (95.3%)	53 (82.8%)		
Diabetes *n* (%)	Yes	17 (26.6%)	16 (25%)	0.041	0.840
	No	47 (73.4%)	48 (75%)		
Osteoporosis *n* (%)	Yes	28 (43.8%)	19 (29.7%)	2.723	0.100
	No	36 (56.3%)	45 (70.3%)		
Anesthesia method *n* (%)	General anesthesia	63 (98.4%)	55 (85.9%)	6.942	0.008
	Lumbar anesthesia	1 (1.6%)	9 (14.1%)		
Surgical site *n* (%)	L	37 (57.8%)	30 (46.9%)	1.535	0.215
	R	27 (42.2%)	34 (53.1%)		
ASA M (P25, P75)		2.000 (1.000, 2.000)	2.000 (1.000, 2.000)	−0.638	0.524
Blood loss volume mL, M (P25, P75)		50.000 (50.000, 100.000)	50.000 (30.000, 100.000)	−0.698	0.485
Operation time (m, ±s)		124.660 ± 25.609	130.250 ± 15.656	−1.491	0.138
Preoperative VAS M (P25, P75)		5.000 (4.000, 6.000)	5.000 (4.000, 6.000)	−0.573	0.567
Preoperative HSS M (P25, P75)		56.000 (52.000, 58.000)	56.000 (52.250, 58.000)	−0.975	0.33
Preoperative Harris M (P25, P75)		55.000 (52.000, 58.000)	55.500 (53.000, 58.000)	−1.224	0.221

**Table 2:** Baseline data of hip joint.

Parameters		Study group	Traditional group	*t*/*Z*/*χ*^2^	*P*
Sex *n* (%)	M	33 (51.6%)	31 (48.4%)	0.125	0.723
	F	35 (54.7%)	29 (45.3%)		
Age (year, *x¯* ± *s*)		55.890 ± 5.207	54.770 ± 5.395	1.2	0.232
BMI (kg/m^2^, *x¯* ± *s*)		25.563 ± 2.941	26.548 ± 3.363	−1.766	0.080
Smoking *n* (%)	Yes	7 (10.9%)	15 (23.4%)	3.513	0.061
	No	57 (89.1%)	49 (76.6%)		
Drinking *n* (%)	Yes	5 (7.8%)	14 (21.9%)	5.006	0.025
	No	59 (92.2%)	50 (78.1%)		
Diabetes *n* (%)	Yes	17 (26.6%)	16 (25)	0.041	0.840
	No	47 (73.4%)	48 (75%)		
Osteoporosis *n* (%)	Yes	33 (51.6%)	26 (40.6%)	1.541	0.215
	No	31 (48.4%)	38 (59.4%)		
Anesthesia method *n* (%)	General anesthesia	61 (95.3%)	58 (90.6%)	1.076	0.300
	Lumbar anesthesia	3 (4.7%)	6 (9.4%)		
Surgical site *n* (%)	L	38 (59.4%)	34 (53.1%)	0.508	0.476
	R	26 (40.6%)	30 (46.9%)		
ASA M (P25, P75)		2.000 (2.000, 2.000)	2.000 (2.000, 2.000)	−0.696	0.487
Blood loss volume mL, M (P25, P75)		50.000 (50.000, 100.000)	50.000 (50.000, 50.000)	−0.698	0.485
Operation time (m, *x¯* ± *s*)		130.000 (111.250, 143.75)	130.000 (115.000, 150.000)	−0.474	0.636
Preoperative VAS M (P25, P75)		5.000 (4.000, 6.000)	4.500 (4.000, 5.000)	−2.104	0.035
					
Preoperative HSS M (P25, P75)		56.000 (52.250, 58.000)	54.500 (52.000, 57.000)	−1.182	0.237

This retrospective cohort study protocol was approved by the Medical Ethics Committee of the 940th Hospital of the Joint Logistics Support Force of the Chinese People's Liberation Army (Approval No. 2025KYLL063). All patients had signed written informed consent for surgery and routine rehabilitation follow-up preoperatively. Since this study only retrospectively analyzed de-identified data from routine clinical care without any additional intervention with patients, the institutional ethics committee formally granted a waiver of informed consent.

## 3 Traditional ERAS interventions for knee and hip joint replacement surgeries

**Preoperative Phase:**

- Psychological counseling and structured health education were provided to alleviate patient anxiety, with clear explanations of the surgical workflow, pain management strategies, and expected rehabilitation goals.
- The preoperative fasting duration was shortened, and patients were allowed to consume oral sugar water 2 h before surgery to reduce the metabolic stress response (7).
- Patients were instructed to maintain appropriate bed rest before surgery, and excessive physical activity was restricted to avoid compromising preoperative physical status.
- General anesthesia combined with peripheral nerve block was administered to reduce postoperative pain sensitivity (8).
- Preoperative anemia correction was performed: for patients with a hemoglobin level <130 g/L, erythropoietin (EPO) combined with intravenous iron sucrose infusion was administered to optimize preoperative hemoglobin levels.

**Intraoperative Phase:**

Strict intraoperative normothermia management was implemented. All intraoperative irrigation fluids were prewarmed to near body temperature to reduce hypothermia-related perioperative complications (8).

**Postoperative Phase:**

- Multimodal analgesia was adopted, with non-steroidal anti-inflammatory drugs as the core regimen, and opioid drugs were supplemented only when necessary to achieve adequate pain relief (9).
- Early oral feeding was initiated: patients were allowed to drink warm water or rice soup 6 h after surgery, then gradually transitioned to a semiliquid diet. The total daily intravenous fluid volume was strictly restricted, and the diet was dominated by high-protein, low-fat, high-fiber food (such as porridge, steamed egg, and vegetable puree).
- The surgical drainage tube was removed once the 24-h postoperative drainage volume was fewer than 100 mL. Urinary function was routinely assessed after surgery, and the urinary catheter was removed as soon as the patient regained full consciousness to reduce the risk of urinary tract infection (9).
- Local soft tissue massage was performed to promote blood circulation and relieve limb swelling. Standardized muscle strength training was initiated: 10 repetitions of quadriceps isometric contractions and ankle pump exercises were performed every hour immediately after the patient regained consciousness.
- Within 24 h after surgery, patients were assisted to stand with a walker, and then gradually progressed to short-distance walking, to prevent deep vein thrombosis and postoperative joint stiffness (9, 10).
- Discharge education was delivered with a detailed home-based rehabilitation plan, including specified daily walking duration and joint range of motion training (such as straight leg raise exercises). Dietary guidance was also provided: a low-salt, low-fat, high-calcium, and high-protein diet was recommended, and weight control was emphasized to reduce mechanical burden on the prosthetic joint.
- Standard outpatient follow-ups were arranged at 1, 3, and 6 months after the surgery to assess the functional recovery.

## 4 Multi-component ERAS intervention for knee and hip arthroplasty

### 4.1 Risk stratification criteria

In this study, a risk stratification scheme based on a multi-dimensional assessment was established by combining an in-house developed prediction model for post-arthroplasty complications from our department with the real-world clinical practice of our center. All patients in the study group completed assessments covering five core dimensions before surgery: smoking status, American Society of Anesthesiologists (ASA) physical status classification, preoperative joint range of motion (ROM), platelet count, and erythrocyte sedimentation rate.

The results of the five predictors were input into the risk prediction model to calculate the corresponding individual risk probabilities, based on which patients were categorized into three risk tiers: low risk (≤30%), moderate risk (31%–70%), and high risk (≥70%). All risk assessments were independently performed by two attending surgeons or senior attending surgeons specialized in joint surgery, and any disagreements were resolved by a third associate chief physician to reach a final consensus. After 1,000 bootstrap resampling iterations, the model yielded a concordance index (C-index) of 0.976 (95%CI: 0.950–0.994). Single-center temporal validation was conducted using independent clinical data from our hospital, and the DeLong test was performed to compare the difference between the area under the curve of the training set (0.976) and that of the validation set (0.987). The result showed *P* = 0.5282 (*P* > 0.05), indicating that the model maintained excellent discrimination performance in both internal and external validation cohorts and demonstrated robust generalizability (Figure 2).

**Figure 2:**
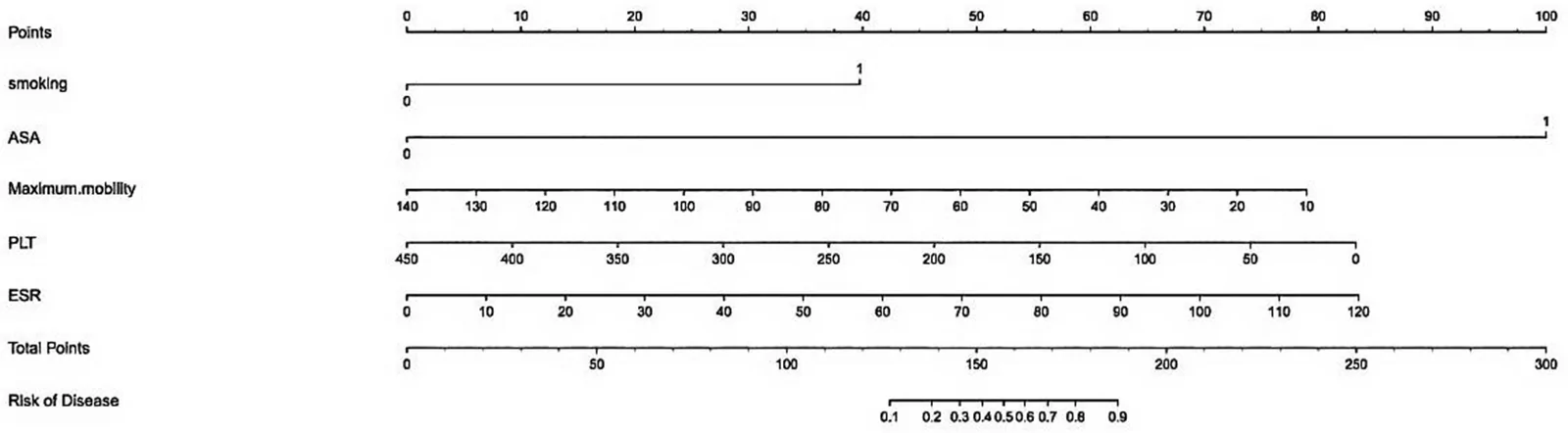
Risk prediction model. Hierarchical assessment indicators: (1) Smoking: No = 0 points, Yes = 1 point; (2) PLT, platelet PLT—actual value; (3) ESR, erythrocyte sedimentation rate—actual value; (4) preoperative joint flexion range of motion—actual value; (5) ASA classification: ≤2 = 0 points, ≥2 = 1 point.

### 4.2 Smart wearable monitoring device and data standardization protocol

Device Specification: The wearable monitoring tool used in this study was the Xiaomi Band 7 (Xiaomi Inc., Beijing, China).

Core Monitored Outcomes: Valid daytime walking steps, accumulated ambulation distance, and repetitive stepping frequency.

Data Extraction and Clinical Access Workflow: At 20:00 on each postoperative day, the attending rehabilitation physician systematically retrieved the de-identified daily activity dataset from the device's cloud-based management platform. These data were uploaded in real time by patients or their authorized family members to avoid manual data entry bias.

Predefined Compliance Control Protocol: The assigned ward nurse performed a daily device connectivity check during routine ward rounds. Patients with a total daily wearing duration of fewer than 12 h were formally defined as non-adherent. There were 13 non-adherent patients in the study group, including eight who underwent knee joint and five who underwent hip arthroplasty.

For both the THA and TKA subgroups in the study group, the baseline training volume for each postoperative phase (acute phase, subacute phase, and late functional recovery phase) was individually calibrated according to the patient's preoperatively assigned low-, moderate-, or high-risk stratification tier. The daily ambulation load metrics captured by the validated smart wearable device served as an objective, irreplaceable reference for rehabilitation physicians to make clinical decisions on whether a patient could safely advance to the next stage of structured rehabilitation training.

## 5 Postoperative rehabilitation therapy for total hip arthroplasty

### 5.1 Acute postoperative phase (postoperative days 1–3)

Appropriate standing training was initiated on the same day the surgical drainage tube was removed. Bedside activities: (a) Ankle pump exercises, quadriceps isometric contractions, gluteal muscle isometric contractions, and hip abduction and extension training; (b) Continuous passive motion (CPM) was initiated on postoperative day 3, with hip flexion strictly limited to <90°, without pain, for 30 min per session, twice daily (11). Posture management: (a) The affected limb was maintained in an abduction neutral position to avoid adduction and internal rotation, and a triangular pillow was placed between the legs during turning (11); (b) Prolonged sitting was strictly restricted (≤30 min per session) to prevent hip flexion from exceeding 90°, and a semi-recumbent position was recommended for rest (11). Ambulation activities (under direct guidance of certified rehabilitation therapists): (a) Standing and walking were performed with the assistance of a walker or crutches, following the “unaffected limb first” principle; (b) Symmetrical lower limb weight-bearing and alternating gait were practiced using the walker (12); (c) Lower limb stationary cycling training (20–30 min per session, twice daily) was conducted; (d) Seated knee flexion training was conducted, with absolute contraindications against hip flexion >90°, hip adduction across the midline, and hip internal rotation beyond the neutral position. Pain management: Oral analgesics and parecoxib sodium injections were administered, combined with local cold compresses (15–20 min per session). Patients with intolerable pain received an additional nerve block for analgesia. Low- and moderate-risk patients completed the full standard training regimen, with adjuvant acupuncture to relieve local soreness. For high-risk patients, all basic rehabilitation exercises were performed in divided low-volume sessions following a strict stepwise progression protocol. Adaptive training was prioritized to maintain a minimum walking volume of 100 m per session, with the entire phase transitioning at a low-load slow pace. Forced volume escalation that might induce swelling and pain, as well as premature asymmetric weight-bearing, was strictly prohibited. A nerve block was provided for patients with poor pain tolerance to support smooth training completion. Progression Criteria: Patients who could complete 200 m of walking and achieve controlled symmetric weight-bearing were allowed to transition from walker-assisted ambulation to crutch-assisted walking.

### 5.2 Mid postoperative phase (postoperative weeks 2–8)

Rehabilitation training from the previous phase was continued, with higher-intensity functional exercises added. (a) Clamshell exercises were performed in the side-lying position with an abduction pillow to enhance the muscle strength of the gluteus medius and hip extensors; (b) Forward step-up exercises were implemented with step heights of 10, 15, and 20 cm for progressive training; (c) Standing hip abduction and extension training was carried out. Low- and moderate-risk patients completed the full progressive training regimen. For high-risk patients, if soreness occurred during step-up training, progression to higher step heights was paused, and only 10 cm step training was maintained. Progression Criteria: Low- and moderate-risk patients who could complete 30 repetitions of marching in place three times a day without soreness and walk 200–500 m without any assistive device, were allowed to progress. Low-risk patients were permitted to briefly reach the upper limit of the training load to accelerate the recovery of gait symmetry. High-risk patients could only advance to the next phase after consecutively meeting the above criteria for two full days.

### 5.3 Late postoperative phase (postoperative weeks 8–14)

Targeted interventions were delivered to address residual impairments identified in functional assessments, including gait imbalance, impaired proprioception, and reduced motor control. (a) Balance training: Standing exercises were performed on a balance pad or soft mat, as well as single-leg standing training (with stable hand support) (13); (b) Gait training: Compensatory abnormal gait patterns were corrected to improve stride length, walking speed, and total ambulation distance; (c) Lateral step training (10–15 cm) (14): The affected limb was placed on the step with the hip flexed at 20°, after which the unaffected foot was slowly lowered off the step and subsequently raised back. At the end of this phase, residual mild lower limb length discrepancy was corrected with a customized heel lift. This intervention helped restore normal hip mechanical alignment and prevented excessive stress on the spine and other weight-bearing joints caused by malalignment (15). Low-risk patients completed the full standard training regimen. Moderate- and high-risk patients could only initiate lateral step training after consecutively completing pain-free walking for over 30 min (3–5 km) and 40 repetitions of marching in place three times a day for two full days. For high-risk patients, proprioception and muscle strength training were prioritized before introducing lateral step exercises (16).

Final Functional Recovery Criteria: Hip range of motion approached normal levels (flexion ≥90°, abduction ≥30°); pain-free walking for over 30 min (3–5 km); and independent completion of activities of daily living, including stair climbing and rising from a seated position.

## 6 Postoperative rehabilitation protocol for total knee arthroplasty

### 6.1 Acute postoperative phase (postoperative days 1–7)

Appropriate standing training was initiated on the same day after surgical drain removal. Bedside Activities: (a) CPM: Range of motion was initially set between −5° and 60° and administered for 40–60 min per day. The flexion angle was gradually increased based on the patient's recovery progress (17). (b) Ankle pump exercises, quadriceps isometric contractions, and gluteal muscle isometric contractions were performed (18). Posture Management: (a) The operated limb was elevated (19). (b) The knee joint was maintained in full extension during nighttime rest to prevent flexion contracture (no pillow was placed under the knee). Ambulation Activities: (a) Gait training with a walker was performed according to the patient's pain tolerance (20). (b) Lower limb stationary cycling training (20–30 min per session, twice daily) was initiated once the surgical wound showed no exudate. Pain Management: (a) Local cold compresses were applied (15–20 min per session) (21). (b) Oral analgesics and parecoxib sodium injections were administered. Patients with intolerable pain received additional nerve block analgesia (22).

Risk-Stratified Implementation: Low-risk patients: Completed CPM and muscle strength training as early as possible. They were encouraged to achieve a single walking distance of 100 m on the first postoperative day, maintaining a stable and normal load. Moderate-risk patients: Started with a short-term, low-load transition (50 m per session). The walking volume was gradually increased only after muscle strength training targets were met. Adjuvant acupuncture was applied to alleviate muscle soreness. High-risk patients: Adhered to a strict, stepwise progression protocol. CPM and muscle strength exercises were performed in divided, low-volume sessions. The initial walking distance was strictly limited to 10–30 m per session to control mechanical load and avoid exacerbating knee swelling. Nerve block analgesia was provided for patients with poor pain tolerance to facilitate exercise completion.

Progression Criteria: Advancement to the mid-term phase was permitted only after patients had completed standardized muscle strength training, achieved a single walker-assisted walking distance of 200 m, and reported no persistent swelling or pain.

### 6.2 Mid postoperative phase (postoperative weeks 2–8)

Joint Range of Motion Training: This included passive knee flexion (using a CPM machine), active knee flexion (seated heel slides), and knee extension training (leg press exercises). Muscle Strength Training: Lower limb stationary cycling training (20–30 min per session, twice daily) was continued. Forward Step-Up Exercises (19): Step heights of 10 cm and 15 cm were used, requiring knee flexion >60°. Balance and Proprioception Training: Exercises included single-leg static standing, bilateral dynamic activities, and standing exercises performed on a balance pad or soft mat. Walking Training: Patients gradually transitioned from a walker to a single crutch cane, or no assistive device while avoiding a limping gait (19). The walking duration was progressively extended to 15–30 min per session.

Risk-stratified implementation: Low-risk patients could rapidly progress to the upper limit of 400–500 m per session. They were allowed to briefly approach the load ceiling and simultaneously initiate step-up and weight-shift training to accelerate gait recovery. Moderate-risk patients: Complete the full regimen of muscle strength training and steadily progressed their walking distance to 400–500 m per session. High-risk patients: Volume escalation was prohibited. The primary focus remained on controlling swelling and managing pain (23).

Progression criteria: Patients were allowed to advance to the next phase after achieving stable walking of 2 km daily for 1 consecutive week, with no reports of soreness or discomfort, a normal gait pattern, and knee flexion greater than 105°.

### 6.3 Late postoperative phase (postoperative weeks 9–16)

Patients continued to reinforce the training regimens from previous phases, including quadriceps and gluteal muscle isometric contraction exercises. The structured training program also incorporated wall squat training, forward step-up exercises (15–20 cm step height), balance and proprioception training, and moderate-to-high-intensity lower limb stationary cycling training (20–30 min per session, twice daily).

Muscle strength training was sustained throughout this period. Patients were strictly advised to avoid high-impact, strenuous activities, such as jumping and contact sports, while low-intensity swimming was recommended as a suitable alternative (24). Weight management was emphasized to reduce mechanical load on the prosthetic knee, and regular outpatient follow-up visits were scheduled to assess prosthesis status. For all low-, moderate-, and high-risk patients, prolonged flat-ground walking was added to the regimen to consolidate muscle strength and improve joint endurance.

The predefined functional recovery criteria were as follows: knee flexion ≥120° with full extension (0°); pain-free walking for more than 30 min (covering 3–5 km) with a natural gait; and independent completion of daily activities including stair climbing and rising from a seated position.

## 7 Statistical analysis

Statistical analyses were performed using SPSS Statistics 27.0 (IBM Corp., Armonk, NY, USA). Normally distributed continuous variables were presented as mean ± standard deviation (*x¯* ± *s*), and an independent-samples *t*-test was applied for between-group comparisons. Non-normally distributed continuous variables were expressed as median with interquartile range M (Q_1_, Q_3_), and a non-parametric rank sum test was used for between-group comparisons. Categorical variables were reported as case numbers and/or percentages, and compared using the Chi-square test or Fisher's exact test. All statistical tests were two-tailed, and a *P*-value <0.05 was defined as statistically significant.

For repeatedly measured outcomes including the Visual Analog Scale (VAS), HSS knee score, and Harris hip score, a generalized estimating equations (GEE) model with a Gaussian distribution, identity link function, and independent working correlation matrix was adopted. The model incorporated the grouping factor, time factor, and group-by-time interaction term, adjusted for all imbalanced baseline variables, and employed robust standard errors. The Bonferroni correction was applied for multiple pairwise comparisons across different follow-up time points. The incidence of postoperative complications was compared using Fisher's exact test.

This study was designed as a retrospective observational study. To control for bias from baseline confounding factors in functional outcome estimates, we included all imbalanced baseline variables as covariates in the GEE model for adjustment, to ensure that the evaluation of intervention effects was not confounded by unbalanced baseline factors. Specifically, the adjusted covariates for TKA included intra-articular injection or acupuncture and anesthesia type, while the adjusted covariates for THA included alcohol consumption history and preoperative hip VAS pain score.

## 8 Results

### 8.1 Baseline characteristics

For the TKA cohort, no statistically significant differences were observed between the study group and the control group in terms of age, BMI, gender distribution, smoking status, alcohol consumption, diabetes mellitus, osteoporosis, surgical site, ASA classification, intraoperative blood loss, operative duration, and preoperative VAS/HSS scores (all *P* > 0.05). However, significant intergroup differences were detected in the history of intra-articular injection/acupuncture and anesthesia type (*P* < 0.05).

For the THA cohort, the two groups were comparable in age, BMI, gender distribution, smoking status, diabetes mellitus, osteoporosis, anesthesia type, surgical site, ASA classification, intraoperative blood loss, operative duration, and preoperative HSS scores (all *P* > 0.05). Statistically significant differences were found in alcohol consumption and preoperative VAS scores between the groups (*P* < 0.05).

All imbalanced baseline variables were incorporated as covariates in the GEE model for adjusted analysis. After adjustment, the adjusted mean differences in HSS scores for the TKA study group were 7.81 (95%CI: 6.43–9.19, *P* < 0.001) at 1 month postoperatively, 8.04 (95%CI: 6.62–9.46, *P* < 0.001) at 3 months, and 4.41 (95%CI: 3.39–5.43, *P* < 0.001) at 6 months. The adjusted intergroup difference trend of VAS scores remained consistent with the unadjusted results (adjusted difference at 1 month: −1.68, 95%CI: −1.91 to −1.45, *P* < 0.001). These findings confirmed that the functional recovery advantage of the study group remained robust after controlling for baseline confounding factors.

For the THA study group, the adjusted mean differences in Harris scores were 5.88 (95%CI: 4.48–7.28, *P* < 0.001) at 1 month postoperatively, 7.65 (95%CI: 6.32–8.98, *P* < 0.001) at 3 months, and 5.01 (95%CI: 3.98–6.04, *P* < 0.001) at 6 months. The adjusted VAS scores in the study group remained significantly lower than those in the control group at all follow-up time points (all *P* < 0.01). These adjusted results further validate the effectiveness of the proposed rehabilitation protocol.

### 8.2 GEE analysis of VAS scores in the TKA cohort

The GEE analysis revealed a significant interaction effect between time and group on VAS scores (Wald *χ*^2^ = 114.268, *P* < 0.001), and subsequent simple effects analysis was performed, indicating distinct postoperative pain trajectory patterns between the two groups.

Intragroup time effect: VAS scores at all postoperative time points in the study group were significantly lower than the preoperative baseline (all *P* < 0.05), showing a continuous improvement trend. In the control group, the VAS score on postoperative day 1 showed no significant difference from the preoperative level (*P* > 0.05), while scores at all other follow-up time points were significantly reduced compared with baseline (all *P* < 0.005) (Table 3).

**Table 3:** Analysis of the within-group time effects of VAS at each follow-up time point before and after surgery in two groups of patients based on GEE (TKA).

	Time	*B*	Standard error	Hypothesis testing			95% Wald CI		Corrected average value	Corrected standard deviation	95% Wald CI after correction	
				Wald *χ*^2^	df	*P*	Lower limit	Upper limit			Lower limit	Upper limit
1	Time 1	−1.625	0.116	196.655	1.000	0.000	−1.852	−1.398	3.440	0.062	3.320	3.560
	Time 2	−3.453	0.119	844.133	1.000	0.000	−3.686	−3.220	1.610	0.061	1.490	1.730
	Time 3	−2.594	0.124	434.380	1.000	0.000	−2.838	−2.350	2.470	0.062	2.350	2.590
	Time 4	−4.453	0.125	1,271.935	1.000	0.000	−4.698	−4.208	0.610	0.061	0.490	0.730
	Time 5	−5.062	0.099	2,640.906	1.000	0.000	−5.256	−4.869	0.000	0.000	0.000	0.000
	Time 0	0										
2	Time 1	0.000	0.142	0.000	1.000	1.000	−0.277	0.277	5.140	0.106	4.930	5.350
	Time 2	−2.687	0.127	449.947	1.000	0.000	−2.936	−2.439	2.450	0.062	2.330	2.580
	Time 3	−1.672	0.106	248.301	1.000	0.000	−1.880	−1.464	3.470	0.062	3.350	3.590
	Time 4	−3.594	0.128	784.430	1.000	0.000	−3.845	−3.342	1.550	0.062	1.420	1.670
	Time 5	−4.547	0.129	1,247.889	1.000	0.000	−4.799	−4.295	0.590	0.061	0.470	0.710
	Time 0	0										

The coefficient represents the parameter estimate relative to the reference level, with Time 0 set as the reference time point. Group 1 = study group; Group 2 = traditional group; Time Time 0 = before surgery; Time 1 = first day after surgery; Time 2 = third day after surgery; Time 3 = one month after surgery; Time 4 = three months after surgery; Time 5 = six months after surgery; VAS: Visual Analogue Scale.

Intergroup comparison: The study group presented significantly lower VAS scores than the control group on postoperative day 1, day 3, 1 month, 3 months, and 6 months (all *P* < 0.005). No significant intergroup difference was detected in preoperative VAS scores (*P* > 0.05) (Table 4).

**Table 4:** Comparison of the inter-group effects of VAS at each follow-up time point before and after surgery in two groups of patients based on GEE (TKA).

Time	Group	*B*	Standard error	Hypothesis testing			95% Wald CI		Corrected average value	Corrected standard deviation	95% Wald CI after correction	
				Wald *χ*^2^	df	*P*	Lower limit	Upper limit			Lower limit	Upper limit
Time 1	Group 1	−1.703	0.123	193.236	1.000	0.000	−1.943	−1.463	3.440	0.062	3.320	3.560
	Group 2	0										
Time 2	Group 1	−0.844	0.087	93.781	1.000	0.000	−1.015	−0.673	1.610	0.061	1.490	1.730
	Group 2	0										
Time 3	Group 1	−1.000	0.088	128.502	1.000	0.000	−1.173	−0.827	2.470	0.062	2.350	2.590
	Group 2	0										
Time 4	Group 1	−0.937	0.087	115.779	1.000	0.000	−1.108	−0.767	0.610	0.061	0.490	0.730
	Group 2	0										
Time 5	Group 1	−0.594	0.061	93.538	1.000	0.000	−0.714	−0.473	0.000	0.000	0.000	0.000
	Group 2	0										
Time 0	Group 1	−0.078	0.141	0.307	1.000	0.580	−0.355	0.198	5.060	0.099	4.870	5.260
	Group 2	0										

The coefficient represents the parameter estimate relative to the reference level, with Time 0 set as the reference time point. Group 1 = study group; Group 2 = traditional group; Time Time 0 = before surgery; Time 1 = first day after surgery; Time 2 = third day after surgery; Time 3 = one month after surgery; Time 4 = three months after surgery; Time 5 = six months after surgery; VAS: Visual Analogue Scale.

### 8.3 GEE analysis of HSS scores in the TKA cohort

A significant time-by-group interaction was identified for HSS scores via GEE analysis (Wald *χ*^2^ = 190.331, *P* < 0.001), and subsequent simple effects analysis was conducted.

Intragroup time effect: HSS scores at all postoperative time points in the study group were significantly higher than the preoperative baseline (all *P* < 0.05). In the control group, the HSS score at 3 days postoperatively showed no statistically significant difference from the preoperative level (*P* = 0.071), while scores at all other time points were significantly elevated compared with baseline (all *P* < 0.05) (Table 5).

**Table 5:** Analysis of the within-group time effects of HSS at each follow-up time point before and after surgery in two groups of patients based on GEE (TKA).

	Time	*B*	Standard error	Hypothesis testing			95% Wald CI		Corrected average value	Corrected standard deviation	95% Wald CI after correction	
				Wald *χ*^2^	df	*P*	Lower limit	Upper limit			Lower limit	Upper limit
1	Time 1	−8.094	0.766	111.642	1.000	0.000	−9.595	−6.592	47.08	0.608	45.89	48.27
	Time 2	7.578	0.640	140.047	1.000	0.000	6.323	8.833	62.75	0.604	61.57	63.93
	Time 3	17.781	0.674	695.162	1.000	0.000	16.459	19.103	72.95	0.546	71.88	74.02
	Time 4	27.750	0.704	1,555.314	1.000	0.000	26.371	29.129	82.92	0.591	81.76	84.08
	Time 5	34.688	0.590	3,461.563	1.000	0.000	33.532	35.843	55.17	0.408	54.37	55.97
	Time 0	0										
2	Time 1	−8.391	0.735	130.411	1.000	0.000	−9.831	−6.951	47.34	0.615	46.14	48.55
	Time 2	−0.906	0.503	3.249	1.000	0.071	−1.892	0.079	62.75	0.604	61.57	63.93
	Time 3	10.359	0.626	273.920	1.000	0.000	9.133	11.586	66.09	0.411	65.29	66.9
	Time 4	19.031	0.627	921.479	1.000	0.000	17.802	20.260	82.92	0.591	81.76	84.08
	Time 5	29.641	0.511	3,367.169	1.000	0.000	28.639	30.642	89.86	0.398	89.08	90.64
	Time 0	0										

The coefficient represents the parameter estimate relative to the reference level, with Time 0 set as the reference time point. Group 1 = study group; Group 2 = traditional group; Time Time 0 = before surgery; Time 1 = first day after surgery; Time 2 = third day after surgery; Time 3 = one month after surgery; Time 4 = three months after surgery; Time 5 = six months after surgery; HSS: hospital for special surgery.

Intergroup comparison: The study group achieved significantly higher HSS scores than the control group at postoperative day 3, 1 month, 3 months, and 6 months (all *P* < 0.001). No significant intergroup difference in HSS scores was observed on postoperative day 1 (*P* > 0.05) (Table 6).

**Table 6:** Comparison of the inter-group effects of HSS at each follow-up time point before and after surgery in two groups of patients based on GEE (TKA).

Time	Group	*B*	Standard error	Hypothesis testing			95% Wald CI		Corrected average value	Corrected standard deviation	95% Wald CI after correction	
				Wald *χ*^2^	df	*P*	Lower limit	Upper limit			Lower limit	Upper limit
Time 1	Group 1	−0.266	0.865	0.094	1.000	0.759	−1.961	1.429	47.080	0.608	45.890	48.270
	Group 2	0										
Time 2	Group 1	6.859	0.684	100.675	1.000	0.000	5.519	8.199	72.950	0.546	71.880	74.020
	Group 2	0										
Time 3	Group 1	7.922	0.718	121.760	1.000	0.000	6.515	9.329	62.750	0.604	61.570	63.930
	Group 2	0										
Time 4	Group 1	8.156	0.732	124.077	1.000	0.000	6.721	9.591	82.920	0.591	81.760	84.080
	Group 2	0										
Time 5	Group 1	4.484	0.538	69.525	1.000	0.000	3.430	5.538	89.860	0.398	89.080	90.640
	Group 2	0										
Time 0	Group 1	−0.562	0.566	0.987	1.000	0.321	−1.672	0.547	55.170	0.408	54.370	55.970
	Group 2	0										

The coefficient represents the parameter estimate relative to the reference level, with Time 0 set as the reference time point. Group 1 = study group; Group 2 = traditional group; Time Time 0 = before surgery; Time 1 = first day after surgery; Time 2 = third day after surgery; Time 3 = one month after surgery; Time 4 = three months after surgery; Time 5 = six months after surgery; HSS: hospital for special surgery.

### 8.4 GEE analysis of VAS scores in the THA cohort

The GEE analysis demonstrated a significant time–group interaction on VAS scores (Wald *χ*^²^ = 139.589, *P* < 0.001), and a simple effects analysis was performed.

Intragroup time effect: VAS scores at all postoperative follow-up time points in both groups were significantly reduced compared with the preoperative baseline (all *P* < 0.05) (Table 7).

**Table 7:** Analysis of the within-group time effects of VAS at each follow-up time point before and after surgery in two groups of patients based on GEE (THA).

	Time	*B*	Standard error	Hypothesis testing			95% Wald CI		Corrected average value	Corrected standard deviation	95% Wald CI after correction	
				Wald *χ*^2^	df	*P*	Lower limit	Upper limit			Lower limit	Upper limit
1	Time 1	−1.516	0.142	114.766	1.000	0.000	−1.793	−1.238	3.520	0.062	3.390	3.640
	Time 2	−3.437	0.127	736.122	1.000	0.000	−3.686	−3.189	1.590	0.061	1.470	1.710
	Time 3	−2.500	0.123	412.903	1.000	0.000	−2.741	−2.259	2.530	0.062	2.410	2.650
	Time 4	−4.422	0.121	1,343.564	1.000	0.000	−4.658	−4.185	0.610	0.061	0.490	0.730
	Time 5	−4.719	0.128	1,362.525	1.000	0.000	−4.969	−4.468	0.310	0.058	0.200	0.430
	Time 0	0										
2	Time 1	0.297	0.128	5.359	1.000	0.021	0.046	0.548	5	5.000	0.106	4.790
	Time 2	−2.156	0.117	337.435	1.000	0.000	−2.386	−1.926	2.55	2.550	0.062	2.420
	Time 3	−1.219	0.124	96.667	1.000	0.000	−1.462	−0.976	3.48	3.480	0.062	3.360
	Time 4	−3.297	0.112	866.852	1.000	0.000	−3.516	−3.077	1.41	1.410	0.061	1.290
	Time 5	−4.187	0.106	1,569.923	1.000	0.000	−4.395	−3.980	0.52	0.520	0.062	0.390
	Time 0	0										

The coefficient represents the parameter estimate relative to the reference level, with Time 0 set as the reference time point. Group 1 = study group; Group 2 = traditional group; Time Time 0 = before surgery; Time 1 = first day after surgery; Time 2 = third day after surgery; Time 3 = one month after surgery; Time 4 = three months after surgery; Time 5 = six months after surgery; VAS, Visual Analogue Scale.

Intergroup comparison: The study group showed significantly lower VAS scores than the control group at preoperative baseline and all postoperative time points (all *P* < 0.05) (Table 8). Although the intergroup difference gradually narrowed over time, the difference remained statistically significant at 6 months postoperatively (*P* = 0.017).

**Table 8:** Comparison of the inter-group effects of VAS at each follow-up time point before and after surgery in two groups of patients based on GEE (THA).

Time	Group	*B*	Standard error	Hypothesis testing			95% Wald CI		Corrected average value	Corrected standard deviation	95% Wald CI after correction	
				Wald *χ*^2^	df	*P*	Lower limit	Upper limit			Lower limit	Upper limit
Time 1	Group 1	−1.484	0.123	145.601	1.000	0.000	−1.725	−1.243	3.52	0.062	3.39	3.64
	Group 2	0										
Time 2	Group 1	−0.953	0.087	118.894	1.000	0.000	−1.124	−0.782	1.590	0.061	1.470	1.710
	Group 2	0										
Time 3	Group 1	−0.953	0.088	116.566	1.000	0.000	−1.126	−0.780	2.530	0.062	2.410	2.650
	Group 2	0										
Time 4	Group 1	−0.797	0.087	84.801	1.000	0.000	−0.966	−0.627	0.610	0.061	0.490	0.730
	Group 2	0										
Time 5	Group 1	−0.203	0.085	5.684	1.000	0.017	−0.370	−0.036	0.310	0.058	0.200	0.430
	Group 2	0										
Time 0	Group 1	0.328	0.148	4.939	1.000	0.026	0.039	0.618	5.030	0.110	4.810	5.250
	Group 2	0										

The coefficient represents the parameter estimate relative to the reference level, with Time 0 set as the reference time point. Group 1 = study group; Group 2 = traditional group; Time Time 0 = before surgery; Time 1 = first day after surgery; Time 2 = third day after surgery; Time 3 = one month after surgery; Time 4 = three months after surgery; Time 5 = six months after surgery; VAS, Visual Analogue Scale.

### 8.5 GEE analysis of Harris scores in the THA cohort

A significant interaction effect between time and group on Harris scores was confirmed by GEE analysis (Wald *χ*^2^ = 1104.697, *P* < 0.001), followed by simple effects analysis.

Intragroup time effect: In the study group, the Harris scores at all postoperative time points were significantly better than the preoperative baseline (*P* < 0.05). In the control group, the Harris scores at all time points except postoperative day 3 were significantly higher than the preoperative baseline (*P* < 0.05, Table 9).

**Table 9:** Analysis of the within-group time effects of Harris at each follow-up time point before and after surgery in two groups of patients based on GEE (THA).

	Time	*B*	Standard error	Hypothesis testing			95% Wald CI		Corrected average value	Corrected standard deviation	95% Wald CI after correction	
				Wald *χ*^2^	df	*P*	Lower limit	Upper limit			Lower limit	Upper limit
1	Time 1	−7.531	0.658	131.113	1.000	0.000	−8.820	−6.242	47.690	0.548	46.610	48.760
	Time 2	6.469	0.669	93.458	1.000	0.000	5.157	7.780	61.690	0.576	60.560	62.820
	Time 3	17.234	0.726	562.858	1.000	0.000	15.811	18.658	72.450	0.578	71.320	73.590
	Time 4	26.813	0.649	1,706.305	1.000	0.000	25.540	28.085	82.030	0.563	80.930	83.140
	Time 5	35.063	0.597	3,449.588	1.000	0.000	33.892	36.233	90.280	0.379	89.540	91.020
	Time 0	0										
2	Time 1	−7.094	0.749	89.716	1.000	0.000	−8.562	−5.626	47.470	0.670	46.160	48.780
	Time 2	1.125	0.619	3.308	1.000	0.069	−0.087	2.337	55.690	0.435	54.830	56.540
	Time 3	10.281	0.633	263.852	1.000	0.000	9.041	11.522	64.840	0.398	64.060	65.620
	Time 4	19.672	0.593	1,099.141	1.000	0.000	18.509	20.835	74.230	0.390	73.470	75.000
	Time 5	30.625	0.515	3,534.131	1.000	0.000	29.615	31.635	85.190	0.365	84.470	85.900
	Time 0	0										

The coefficient represents the parameter estimate relative to the reference level, with Time 0 set as the reference time point. Group 1 = study group; Group 2 = traditional group; Time Time 0 = before surgery; Time 1 = first day after surgery; Time 2 = third day after surgery; Time 3 = one month after surgery; Time 4 = three months after surgery; Time 5 = six months after surgery; Harris, Harris Hip Score.

Intergroup comparison: The study group obtained significantly higher Harris scores than the control group at postoperative day 3, 1 month, 3 months, and 6 months (all *P* < 0.05). No statistically significant intergroup difference in Harris scores was detected on postoperative day 1 (*P* > 0.05, Table 10).

**Table 10:** Comparison of the inter-group effects of Harris at each follow-up time point before and after surgery in two groups of patients based on GEE (THA).

Time	Group	*B*	Standard error	Hypothesis testing			95% Wald CI		Corrected average value	Corrected standard deviation	95% Wald CI after correction	
				Wald *χ*^2^	df	*P*	Lower limit	Upper limit			Lower limit	Upper limit
Time 1	Group 1	0.219	0.866	0.064	1.000	0.801	−1.479	1.916	47.690	0.548	46.610	48.760
	Group 2	0										
Time 2	Group 1	7.609	0.702	117.393	1.000	0.000	6.233	8.986	72.450	0.578	71.320	73.590
	Group 2	0										
Time 3	Group 1	6.000	0.722	69.115	1.000	0.000	4.585	7.415	61.690	0.576	60.560	62.820
	Group 2	0										
Time 4	Group 1	7.797	0.685	129.457	1.000	0.000	6.454	9.140	82.030	0.563	80.930	83.140
	Group 2	0										
Time 5	Group 1	5.094	0.526	93.661	1.000	0.000	4.062	6.125	90.280	0.379	89.540	91.020
	Group 2	0										
Time 0	Group 1	0.656	0.543	1.459	1.000	0.227	−0.408	1.721	55.220	0.387	54.460	55.980
	Group 2	0										

The coefficient represents the parameter estimate relative to the reference level, with Time 0 set as the reference time point. Group 1 = study group; Group 2 = traditional group; Time Time 0 = before surgery; Time 1 = first day after surgery; Time 2 = third day after surgery; Time 3 = one month after surgery; Time 4 = three months after surgery; Time 5 = six months after surgery; Harris, Harris Hip Score.

### 8.5 Postoperative complications

Postoperative complications in the two groups were recorded and followed up throughout the study period. In the TKA cohort, two cases of DVT and two cases of SSI occurred in the study group, while three DVT cases and three SSI cases were reported in the control group. In the THA cohort, the study group had two DVT cases and one SSI case, whereas the control group had three DVT cases and three SSI cases. No statistically significant differences in the incidence of DVT or SSI were found between the study group and the control group in either the TKA or THA cohorts (all *P* > 0.05). Only a marginal decreasing trend in adverse event counts was observed in the study group (Table 11).

**Table 11:** Comparison of the occurrence of deep vein thrombosis and infection complications in the lower extremities of the two groups of patients [*n* (%)].

Group	Knee (*n* = 128)		Hip (*n* = 128)	
	DVT	SSI	DVT	SSI
Study group	2 (3.13)	2 (3.13)	2 (3.13)	1 (1.56)
Traditional group	3 (4.69)	6 (9.38)	3 (4.69)	3 (4.69)
*P* ^a^	0.648	0.144	0.648	0.309

a The Fisher exact probability method is used for the test; DVT was confirmed by color Doppler ultrasound of the lower extremity vessels; SSI was defined according to the standards of the US CDC.

### 8.6 Results of sensitivity analysis

In all sensitivity analysis scenarios for knee joint indicators (VAS, HSS), the 95% confidence intervals of the between-group mean differences did not contain the null value, and all *P* values were <0.05, with consistent direction and significance, indicating robust and reliable results. The hip Harris score also showed good robustness, with statistically significant between-group differences under all strategies. However, the hip VAS pain score was sensitive to missing data: the between-group difference was significant in the primary analysis and the best-case scenario (*P* < 0.05), while the 95% confidence interval crossed the null value (−0.235–.091, *P* = 0.387) in the worst-case scenario, and the difference was no longer statistically significant. This result suggests that the between-group comparison of hip VAS pain score is sensitive to missing data, showing no statistically significant difference (Table 12).

**Table 12:** Results of sensitivity analysis for the two groups of TKA and THA.

Surgical procedure	Outcome index	Analysis method	Standard deviation	95%CI	*P*
TKA	VAS	Primary analysis	0.061	(−0.714, −0.473)	<0.001
		Worst-case scenario	0.074	(−0.535, −0.245)	<0.001
		Best-case scenario	0.059	(−0.733, −0.502)	<0.001
	HSS	Primary analysis	0.538	(3.430, 5.538)	<0.001
		Worst-case scenario	0.691	(0.899, 3.609)	<0.001
		Best-case scenario	0.545	(4.600, 6.737)	<0.001
	VAS	Primary analysis	0.085	(−0.37, −0.036)	0.017
THA		Worst-case scenario	0.083	(−0.235, 0.091)	0.387
		Best-case scenario	0.079	(−0.432, −0.122)	<0.001
	Harris	Primary analysis	0.526	(4.062, 6.125)	<0.001
		Worst-case scenario	0.691	(1.654, 4.363)	<0.001
		Best-case scenario	0.531	(5.051, 7.131)	<0.001

The primary analysis uses the dataset with missing data excluded; the worst-case scenario adopts the following imputation strategy: the VAS scores in the study group are imputed with the maximum value, while HSS and Harris scores are imputed with the minimum value; the VAS scores in the traditional group are imputed with the minimum value, while HSS and Harris scores are imputed with the maximum value; the best-case scenario adopts the following imputation strategy: the VAS scores in the study group are imputed with the minimum value, while HSS and Harris scores are imputed with the maximum value; the VAS scores in the traditional group are imputed with the maximum value, while HSS and Harris scores are imputed with the minimum value.

## 9 Discussion

The global volume of total joint arthroplasties is increasing. To meet this growing demand, there has been a sustained focus on accelerating patient functional recovery, culminating in the development of ERAS pathways (25). With the rapidly aging population in China and worldwide, the number of THA and TKA cases have risen sharply. Developing practical strategies to improve healthcare quality and provide better outcomes for patients undergoing THA and TKA. While traditional ERAS pathways have been reported to promote early recovery and benefit patients, challenges such as significant individual variability and insufficient standardization in postoperative functional recovery remain. Conventional ERAS, due to its lack of accurate assessment of patients' functional status, suffers from drawbacks such as suboptimal exercise intensity and prolonged duration, failing to reflect physiological function and consequently impacting rehabilitation efficiency (26). Postoperative issues, including pain, swelling, limited joint range of motion, muscle weakness, diminished proprioception, and reduced joint stability, pose substantial barriers to lower limb functional recovery and independence in daily activities (9, 27, 28). To identify rehabilitation problems early in the perioperative course, it is essential to conduct thorough rehabilitation assessments, pinpoint specific issues, and formulate targeted rehabilitation plans and treatments. This is significant for improving joint replacement outcomes and accelerating patient recovery, representing an improvement and refinement of the ERAS concept.

This retrospective cohort study analyzed data from 256 patients undergoing knee or hip arthroplasty, comparing postoperative functional recovery between a multicomponent integrated ERAS pathway—which incorporated preoperative risk stratification and dynamic rehabilitation adjustments—and a traditional ERAS protocol, rather than the efficacy of a single intervention. The inherent nature of retrospective research dictates that causal inferences should be made cautiously; these findings represent statistical associations rather than causal effects.

In the TKA cohort, the postoperative VAS pain scores in the study group were significantly lower than those in the control group at all follow-up time points (Table 4). The sensitivity analysis results showed that under extreme missing-data imputation (worst-case and best-case scenarios), the intergroup difference in VAS scores in the TKA cohort remained statistically significant with consistent direction, indicating that the pain improvement effect in TKA was not interfered with by missing data, and the conclusion was robust. In the THA cohort, although baseline preoperative VAS scores differed between the two groups, the postoperative VAS scores in the study group were significantly lower than those in the control group at all follow-up time points after GEE adjustment. Sensitivity analyses showed that when missing data were imputed under the worst-case scenario, the 95% confidence interval for the intergroup difference in THA–VAS crossed the null value, and the statistical difference disappeared. This indicates that the pain benefit in the THA cohort is highly sensitive to missing data; its statistical significance may depend on bias control of complete data, and clinical interpretation should be cautious. However, this also highlights that objective functional indicators have greater value in evaluating stratified rehabilitation effects.

In contrast to the heterogeneous robustness performance of pain indicators, functional scores remained highly consistent across all sensitivity scenarios, forming the most reliable core finding of this study: For the HSS scores in the TKA cohort: the study group achieved a significant intragroup improvement on postoperative day 3 (*B* = 7.578, *P* < 0.001), while the control group showed no significant improvement on postoperative day 3 compared with the preoperative level (*B* = −0.906, *P* = 0.071), and the first significant improvement did not appear until 1 month postoperatively (*B* = 10.359, *P* < 0.001). The intergroup difference peaked at 8.156 points at 3 months postoperatively. The sensitivity analysis results showed that even under the worst-case scenario, the intergroup difference of TKA–HSS still had statistical significance (*P* < 0.001), and neither the direction nor the significance was reversed. Harris score in THA cohort: The control group showed no significant improvement on postoperative day 3 compared with the preoperative level (Table 9, *B* = 1.125, *P* = 0.069), and achieved significant improvement until 1 month postoperatively (*B* = 10.281, *P* < 0.001); whereas the group was significantly better than the preoperative level on postoperative day 3 (*B* = 6.469, *P* < 0.001). The intergroup difference reached a peak of 7.797 points at 3 months postoperatively. The sensitivity analysis also showed that the intergroup difference in the THA–Harris scores remained statistically significant under the worst-case scenario (*P* < 0.001). These two functional indicators remained statistically significant under the extreme missing assumption, indicating that even with the most unfavorable extreme imputation of missing data, the core conclusion of this study that “the stratified protocol is significantly associated with better postoperative function” is still stable, and there is no risk of conclusion reversal caused by missing data. This methodological verification enhances the credibility of the evidence as a reference for clinical decision-making.

The functional plateau observed in the control group at this juncture suggests that a uniform, low-intensity training model without stratification may be insufficient to counteract early postoperative joint stiffness. In contrast, the study group, by employing risk stratification to advance low- and moderate-risk patients earlier to higher-intensity rehabilitation such as muscle strength training, stationary cycling, and step training, demonstrated a superior functional improvement trend at this critical 1-month mark. This indicates that the stratified rehabilitation protocol was statistically associated with an earlier functional turning point, potentially helping to shorten the early postoperative functional plateau.

Although the number of DVT and SSI cases in the study group was numerically lower, there was no statistically significant difference between the two groups. Limited by the number of adverse events, this study cannot confirm that the safety of this stratified rehabilitation protocol is equivalent or superior to that of the conventional ERAS protocol, and this numerical trend can only serve as a preliminary clinical signal. In terms of clinical significance evaluation criteria, there is currently no universally recognized threshold to judge whether the incremental benefit of the novel rehabilitation regimen over the control regimen reaches a level of clinical perceptibility. For future standardized assessment of intergroup incremental benefits, dedicated anchor-based studies are recommended to establish an MCID reference system applicable to intergroup comparisons. This study further expands the scope of ERAS by introducing a preoperative risk prediction model and smart device monitoring, facilitating a transition from “empirical rehabilitation” to “data-driven rehabilitation” (29).

### 9.1 Interpretation of intergroup differences

The existence of the aforementioned statistical associations can be explained through multiple, non-mutually exclusive pathways: First, the “matching-intensity” optimization effect of preoperative risk stratification played a central role. The study group utilized a risk prediction model to categorize patients into three risk levels, implementing differentiated rehabilitation intensities accordingly—earlier progression to higher-intensity training for low-risk patients and a “low-volume, fractionated, stepwise” strategy for high-risk patients. This “risk-intensity matching” may enable more patients to achieve adequate training volume safely, thereby optimizing the functional recovery trajectory, particularly during the critical 1-month postoperative window for functional breakthrough. Second, the combined effect of the multicomponent bundled protocol was likely contributory. Compared with the control group, the study group received a comprehensive, synchronously adjusted pathway: preoperative risk stratification, smart device monitoring, individualized rehabilitation intensity adjustment, stepwise ambulation goals, and more frequent rehabilitation assessments (29–31). The intergroup differences cannot be attributed to any single component but are more likely the result of synergistic effects among multiple components. While smart device monitoring itself may not directly improve function, it provides objective activity data, enabling rehabilitation physicians to make more precise progression decisions.

The findings of this study should be interpreted as demonstrating a statistical association between a multicomponent, integrated ERAS pathway incorporating rehabilitation stratification assessment and better short-term outcomes following TKA/THA. This association may stem from the precise matching effect of assessment-based stratification and the combined action of multiple synergistic components. This result aligns with previous ERAS research indicating that perioperative multidisciplinary collaboration and standardized management can shorten hospital stays and improve patient functional outcomes (32).

## 10 Limitations

Nevertheless, this study has several limitations. First, the study group received a multi-component, synchronously adjusted comprehensive pathway compared to the control group, making it impossible to attribute the observed differences to any single component. Second, the risk prediction model used in this study demonstrated good discriminative ability through internal validation with 1,000 bootstrap resamples and Single-center temporal validation within our institution; it was developed and validated entirely based on data from our single center and has not undergone prospective validation in external, independent medical centers. Third, this study employed a historical control design, with patients from the two groups enrolled in different time periods. Although all patients were treated by the same surgical team within the same nursing unit, the potential influence of the natural evolution of perioperative management standards over time on outcomes cannot be entirely excluded. To partially mitigate this bias, we adjusted for all measurable baseline confounding factors in the GEE model. However, it is undeniable that certain unmeasured time-varying confounders (e.g., surgeon learning curve, nursing proficiency) may still exist. Future prospective randomized controlled trials are the most reliable way to validate these findings. Fourth, the applicability of this protocol to complex patients, such as the elderly, those with multiple comorbidities, or those with cognitive impairment, remains unclear. Fifth, the Xiaomi Band 7 used in this study is a consumer-grade device, and its step-count measurement may be inaccurate. Future studies will use validated, medical-grade devices for calibration. The conclusions of this study should be strictly considered as evidence of association. These limitations suggest that clinical use of this protocol requires caution. Subsequent confirmatory studies—prospective, multicenter, randomized controlled, with precalculated sample sizes, assessor blinding, and long-term follow-up (≥2 years)—are necessary to clarify the true efficacy boundaries and applicability of this stratified rehabilitation protocol.

## Data Availability

The original contributions presented in the study are included in the article/Supplementary Material; further inquiries can be directed to the corresponding author.
